# Nonrandom filtering effect on birds: species and guilds response to urbanization

**DOI:** 10.1002/ece3.2144

**Published:** 2016-05-03

**Authors:** Carmen Paz Silva, Roger D. Sepúlveda, Olga Barbosa

**Affiliations:** ^1^ Instituto de Ciencias Ambientales y Evolutivas Facultad de Ciencias Universidad Austral de Chile Valdivia Chile; ^2^ Instituto de Ecología y Biodiversidad (IEB‐Chile) Santiago Chile; ^3^ Centro de Desarrollo Urbano Sustentable (CEDEUS) Santiago Chile

**Keywords:** Birds, diversity, evenness, homogenization, nonrandom loss, urban

## Abstract

Using bird survey data taken in three cities in Southern Chile, we evaluated the hypothesis that changes in community composition from periurban to urban areas are not random. Furthermore, the consistency of species and guild loss was assessed across cities. A consistent pattern of difference in community and guild structure between urban and periurban habitats was found. In addition, a nonrandom loss of species was found in urban areas compared to periurban areas, and non‐native species dominated urban communities in all cities. The average abundance of omnivores, granivores, and habitat generalists was higher in urban areas, while insectivores and open habitat species were more abundant in periurban areas. These results strongly suggest that urban habitats act as filters offering suitable conditions for only a fraction of the bird species present in a given area, and the lack of suitable conditions may be facilitating local biotic homogenization in the three studied cities. The results of this study not only fill a biogeographical knowledge gap, but the work presented here also aids the general understanding of factors that affect community structure in habitats with varied levels of local and global urbanization.

## Introduction

Urbanization, understood as the transition of undeveloped land to built‐up or sealed areas (Berland 2012), is proceeding at a fast rate in many parts of the world and notably so in developing countries (United Nations 2014). Urbanization has been recognized as the most pervasive and extreme form of habitat transformation (Alberti 2005; Sochat et al. 2010), being biotic homogenization one consequence of this transformation (Olden et al. 2004; McKinney 2008).

Many fast‐growing cities are often located in regions with high levels of biodiversity (Liu et al. 2003; Pandit and Laband 2007). For example, all of the 35 recognized biodiversity hotspots contain urban areas (Cincotta et al. 2000; Mittermeier et al. 2011), highlighting the importance of understanding the underlying causes of species loss due to urbanization.

Despite the inherent differences among cities, urban areas are designed to fulfill human needs, both functionally and aesthetically. As a result, similar urban environments can be found worldwide, and these habitats are often less suitable for a large fraction of species, while favorable conditions are created for few species that are able to adapt to these new conditions (Evans et al. 2011; Sih et al. 2011). The nonrandom loss of diversity and biotic homogenization are two processes associated with the differential response of species to urban ecosystems. Nonrandom loss of diversity occurs because species that share particular traits (or the lack of them) may be more vulnerable to extinction (Sol et al. 2014). Biotic homogenization is defined as the increase in the similarity of the biota composition between two or more regions over time by the replacement of native localized species with exotic, widespread, and cosmopolitan species (McKinney and Lockwood 1999; Olden and Rooney 2006). Both processes have profound consequences; the simplification of communities can lead to ecosystems with a diminished resilience to environmental change (Olden et al. 2004; van Rensburg et al. 2009) and an increased vulnerability to species invasions (Lyons and Schwartz 2001; Marvier et al. 2004). Furthermore, the simplification of communities can affect ecosystem functions and ecosystem services, therefore directly impacting human well‐being (Olden et al. 2004).

Studies using birds as model organisms have provided evidence supporting the differential response to urbanization (Croci et al. 2008; Evans et al. 2011; Sol et al. 2014). Habitat generalists, omnivores, seedeaters, and widely distributed species are favoured by urbanization (Croci et al. 2008; Evans et al. 2011). In contrast, habitat and food specialists, ground nesters, and primary cavity excavators are negatively affected by urbanization (van Turnhout et al. 2010; Conole and Kirkpatrick 2011).

However, the majority of this knowledge has been derived from studies in developed countries in Europe and North America. In contrast, little is known about developing regions such as Latin America, although this region is experiencing rapid urbanization (United Nations, Department of Economic and Social Affairs, Population Division 2014). For example, a recent global review on the effects of urbanization on bird and plant diversity (Aronson et al. 2014) presented information for 147 cities around the world, yet only three cities in Latin America (LA) were included. This is especially worrisome given that many LA cities largely span six biodiversity hotspots (Pauchard and Barbosa 2013). Consequently, many authors have remarked on the importance of increasing research in cities of developing countries (Leveau and Leveau 2004; Gaston 2010; Ortega‐Álvarez and MacGregor‐Fors 2011).

This aim of this study was to evaluate whether bird communities are affected by nonrandom filtering in three rapidly developing cities in the Chilean Winter Rainfall‐Valdivian Forest biodiversity hotspot (Mittermeier et al. 2011). This question was addressed by testing whether (1) the pattern of change in community composition from periurban to urban areas is a random process and (2) there are consistent patterns of loss of species and guilds across cities. The results of this study not only fill a biogeographical knowledge gap, but also contribute to understanding whether the effects on community structure can be generalized even when there is a variation in urbanization at local and global levels.

## Methods

### Study sites

**Figure 1:**
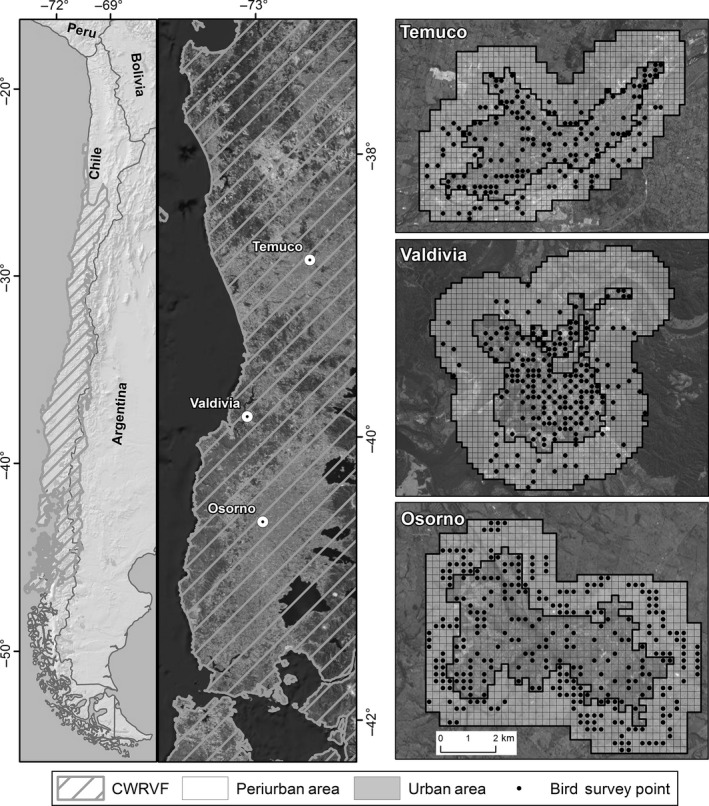
Map of Chile showing the location of Temuco, Valdivia, and Osorno and details of each city including limits of the urban and periurban habitats. Bird surveys were conducted during the breeding season of 2012. The following abbreviation is used: CWRVF, Chilean Winter Rainfall‐Valdivian Forest.

Research was conducted in three cities in Southern Chile, Temuco (38°46′S, 72°38′W), Valdivia (39°48′S, 73°14′W), and Osorno (40°36′S, 73°4′O) (Fig. 1). These cities are categorized as medium‐sized cities according to the Ministerio de Vivienda y Urbanismo (MINVU, 2007). They have sprawling development (UN HABITAT, 2012; Pauchard and Barbosa 2013) and house populations of 100,000–350,000 inhabitants, concentrating 29% of the Chilean population (INE, 2002). All three cities are within the Chilean Winter Rainfall‐Valdivian Forest biodiversity hotspot and are in the Valdivian Rain Forest ecoregion. This region has a prevailing wet temperate climate (Di Castri and Hayek 1976) with less rainy austral summers (December–March); the rainfall values throughout the year range from 1100 to 1900 mm.

### Spatial analysis unit

Using Geographic Information Systems (GIS), a spatially explicit database was generated for the three urban areas and their surrounding landscape matrices. Using the Fishnet application within ArcGis^®^ 9.3 (ESRI), a grid was created with equally sized cells (Barbosa et al. 2007). Cell sizes of 250 × 250 m were selected to allow for an adequate resolution to detect the differences between cells when using a high‐definition digital image (0.5‐m multispectral Geoeye images were acquired in April 2010 for Valdivia and in April 2011 for Temuco and Osorno). Urban areas are generally defined by local administrative boundaries. To enable the comparisons between cities, we defined the urban limit of each city by considering all grid cells that are both within the administrative borders of each city and that have a building coverage of ≥12.5% (Silva et al. 2015). In addition, the periurban area was defined by creating a 1‐km buffer from the urban area limits previously defined as shown in Figure 1. This resulted in a total study area of 67 km^2^ for Temuco (27 km^2^ urban and 40 km^2^ periurban), 62 km^2^ for Valdivia (27 km^2^ urban and 35 km^2^ periurban), and 58 km^2^ for Osorno (25 km^2^ urban and 33 km^2^ periurban).

Vegetation cover was calculated using supervised classification and the normalized difference vegetation index (NDVI). The grid cells where a significant urban development occurred after digital image acquisition were updated accordingly using architectural plans from the municipal council of the three cities.

### Bird surveys and guild classification

We selected 110 urban cells from each city using a stratified random sampling technique; building density was used as a proxy to estimate the degree of urbanization, and 50 periurban cells were chosen randomly in the 1‐km buffer previously defined for each city. Surveys were conducted in each of the grid cells to evaluate species richness and the relative abundance of the bird community. A fixed 50‐m‐radius point‐count methodology (Bibby et al. 2003) was used to register all birds seen or heard over the course of 6 min (Willson et al. 1994; Vergara and Armesto 2009; Silva et al. 2015). The centroid of each grid cell was used as the sampling point. If the point was inaccessible due to it being on private property, the nearest point available within the same grid cell was chosen and recorded using a Garmin Etrex 30 GPS unit. Surveys were conducted during the breeding season of 2012 (September–December) within 4 h after dawn. Each point was surveyed one time. To prevent overlapping observations of bird individuals, the minimal distance between survey points was 250 m (Díaz et al. 2005). Surveys were not performed during rainy days or when wind was strong enough to be a potential impediment to accurate identification. Bias associated with bird detectability was assumed to be constant because the number of bird species present in the cities and surrounding areas is small with <45 possible species (Díaz 2005). All species are easy to recognize and were surveyed at fairly close range (50 m or less). All bird species recorded were classified into 11 guilds corresponding to two resource groups – diet and preferred habitat. Classifications were based on the descriptions provided by Elsam (2006), Jaramillo (2005), and Schulenberg et al. (2007). Species were classified into one of the mutually exclusive guilds for both resource groups; this was performed considering the reported predominant diet and habitat. Guilds considered for diet were carnivore, carrion, frugivore, granivore, insectivore, nectarivore, and omnivore. For the preferred habitat, birds were classified into four guilds: forest, open, water/wetland, and generalist (see Appendix S1 for details).

### Data analysis

To evaluate whether urban bird communities are randomly structured, for each urban location 999 random communities were generated by randomly drawing (with replacement) individuals from a pool of species that occurred in the same proportion as in the surrounding community (Sol et al. 2014). To simplify the comparisons and to avoid confounding random dispersal with community size, the simulated communities had the same community size as the corresponding urban community. A species was considered to be absent in the urban habitat as a result of random filtering if its abundance in all the simulations had a mode of zero (Sol et al. 2014). Species loss analysis was implemented in the software R (R Development Core Team, 2011) using the package vegan (Oksanen et al. 2011) following Sol et al. (2014).

Species richness (the number of species), relative abundance (log‐evenness index), and whole avian community structure (fourth‐root‐transformed data) were compared separately through permutational multivariate analyses of covariance (two‐way PERMANCOVA) using “habitat” (urban and periurban) as a fixed factor, “city” (Temuco, Valdivia, and Osorno) as a random factor, and vegetation cover as a covariate. Preferred habitat (forest, open, water/wetland, and generalist) and diet guilds (carnivore, carrion, frugivore, granivore, insectivore, nectarivore, and omnivore) were examined following the same procedure, but only the functional community structure was considered. Probability values (*P*_perm_) were derived from a pseudo‐F distribution calculated using 10,000 permutations of the same data pool. When the simulated permutations were <1000, probability values were obtained through Monte Carlo simulations (P_MC_). Principal coordinate analysis (PCoA) ordination plots based on centroidal dissimilarity matrices were performed to visualize the size effects of habitats and cities on the bird community structure, preferred habitats, and diet guilds (Clarke and Gorley 2006). The relative contribution of each taxon to the spatial variation in bird community structure was determined through similarity percentage routines (SIMPER, Clarke and Warwick 2001). In this procedure, Bray–Curtis dissimilarities were estimated between and within habitats and cities for the entire dataset of species abundance. The average between‐group dissimilarities were then broken down (cutoff value of 90%) to obtain the highest contributions. PERMANCOVA and SIMPER analyses were performed using PERMANOVA+ for the PRIMER statistical package (Clarke and Gorley 2006).

## Results

**Figure 2:**
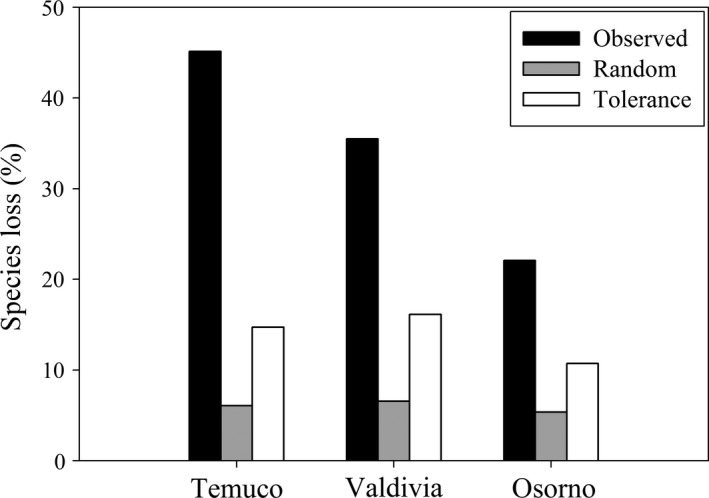
Observed and expected diversity loss due to urbanization in each city. The observed loss is the percentage of periurban habitat species absent in the urban habitat. The random loss is the percentage of the species lost due to random dispersion (i.e., difference between average expected richness based on the simulated urban communities in relation to the recorded species richness in the periurban habitat). Tolerance is the expected richness if all the species classified as avoiders and not observed in the urban habitat are removed from the total recorded species.

The simulations revealed that the loss of species expected by chance was low compared to the loss that was actually detected (Fig. 2). Fifty‐one native species and three introduced bird species were recorded during this study (Appendix S2). Only 16 species were present in all the sample sites, three were present exclusively in urban sites, and 17 were only found in periurban areas. For example, forest specialist species such as Chucao and Magellanic Tapaculo and open habitat species such as Fire‐eyed Diucon and Patagonian Sierra‐finch were only found in periurban areas. The most abundant species in the three urban areas were the House Sparrow and the Chilean Swallow, while the Southern Lapwing was the most abundant species in all three periurban areas.

The influence of vegetation cover was significant on species richness, evenness, community structure, and diet and preferred habitat guilds; the components of variation explained 8.97% and 14.70% of the total variation (Table 1). In all cases, vegetation cover had a significant interaction with city and habitat (Table 1).

**Table 1:** PERMANCOVA outputs for (a) species richness (species number), relative abundance (evenness index) (both on Euclidean distances matrices), and community structure (Bray–Curtis similarity matrix), and for (b) diet and preferred habitat guild structures (Bray–Curtis similarity matrices) of the bird communities, using “habitat” (urban and periurban) as a fixed factor, “city” (Temuco, Valdivia, and Osorno) as a random factor, and “vegetation cover” as a covariate

(a)	*df*	Species number			Evenness index			Community structure		
Source of variation		_pseudo_ *F*	*P*_perm_	CV	_pseudo_ *F*	*P*_perm_	CV	_pseudo_ *F*	*P*_perm_	CV
Vegetation cover	1	53.27	<0.001	14.70	30.28	<0.001	8.97	53.06	<0.001	12.34
City	2	13.26	<0.001	4.62	10.02	<0.001	4.37	7.98	<0.001	2.95
Habitat	1	1.56	0.325^a^	1.64	18.13	0.052^a^	2.38	10.67	0.003^a^	11.39
Vegetation cover × City	2	7.03	0.001	2.45	4.41	0.014	1.77	1.05	0.416	0.02
Vegetation cover × Habitat	1	10.00	0.003	8.30	12.28	0.001	6.24	8.36	<0.001	4.90
City × Habitat	2	4.97	0.008	6.85	0.18	0.824	0	1.78	0.053	1.50
Vegetation cover × City × Habitat	2	1.94	0.135	2.08	0.99	0.345	0	1.14	0.343	0.34
Residual	487			59.36			76.27			66.56

(b)	*df*	Diet guilds			Habitat guilds		
Source of variation		_pseudo_ *F*	*P*_perm_	CV	_pseudo_ *F*	*P*_perm_	CV
Vegetation cover	1	68.74	<0.001	11.85	47.00	<0.001	11.21
City	2	4.48	0.005	1.39	7.24	0.001	2.84
Habitat	1	16.52	0.027^a^	7.64	1.65	0.290^a^	2.67
Vegetation cover × City	2	4.16	0.006	1.36	2.54	0.091	0.75
Vegetation cover × Habitat	1	22.04	<0.001	10.98	1.80	0.202	0.97
City × Habitat	2	0.79	0.518	0	5.71	0.004	9.82
Vegetation cover × City × Habitat	2	2.65	0.055	3.88	0.34	0.715	0
Residual	487			62.91			71.74

a Probability values were derived from Monte Carlo simulations (P_MC_). CV = components of variance (%).

The interaction between habitat and city was significant for species number (_pseudo_ *F*_(2,487)_ = 4.97; *P*_perm_ = 0.008), but nonsignificant for evenness (_pseudo_ *F*_(2,487)_ = 0.18; *P*_perm_ = 0.824), although the city showed an individual effect on the relative abundance (Table 1a). These results indicate that the variation in species number among urban and periurban habitat is different between cities, while relative abundance between habitats is similar in different cities.

**Figure 3:**
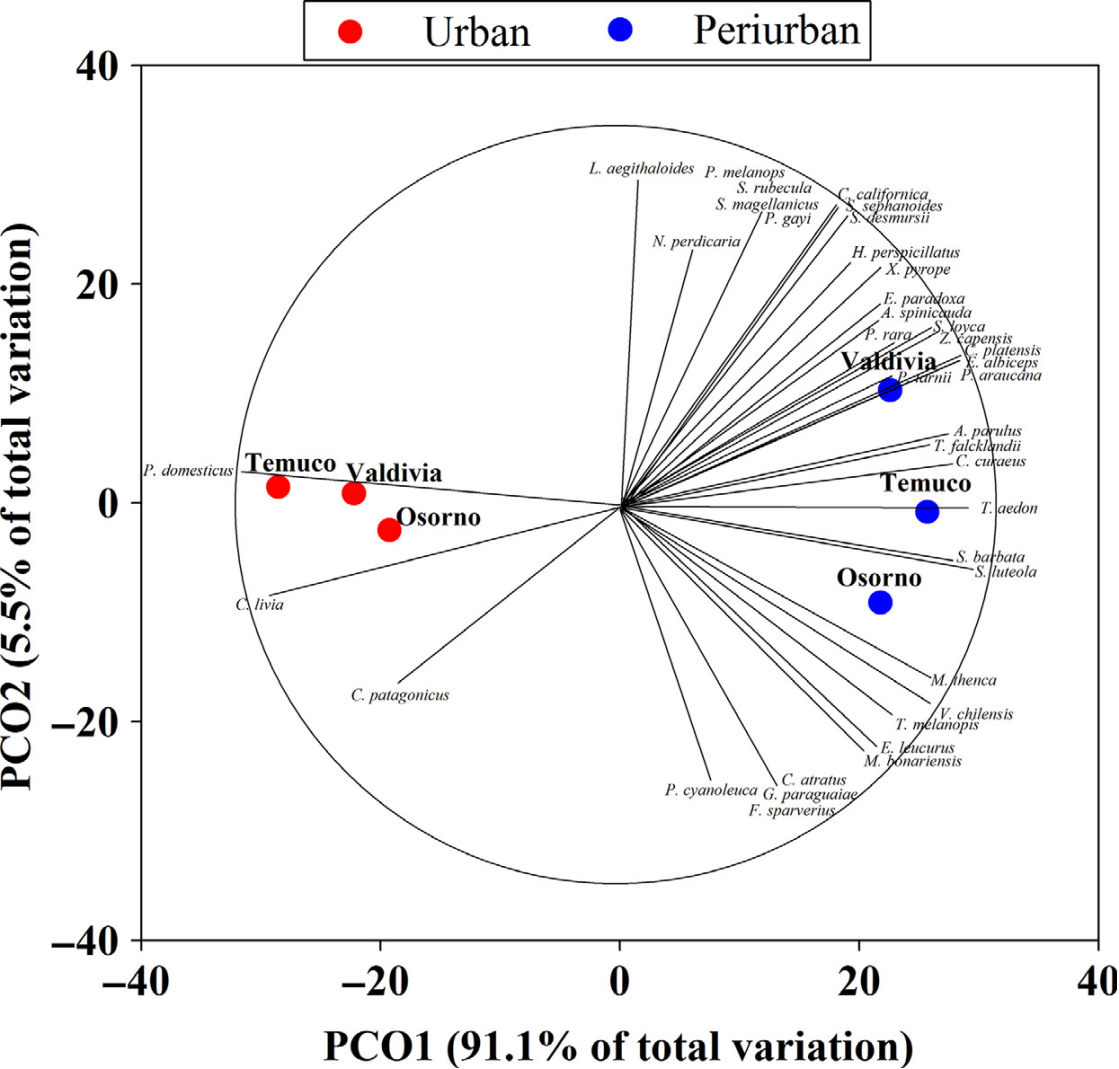
Principal coordinate (PCoA, Bray–Curtis dissimilarity matrices) ordination plot of the bird community structure between urban and periurban habitats for the Temuco, Valdivia, and Osorno. Vectors indicate the correlations >0.75 for each species.

The interaction between city and habitat had a marginally nonsignificant effect on community structure (_pseudo_ *F*_(2,487)_ = 1.78; *P*_perm_ = 0.053) (Table 1a), indicating that the specific composition of habitats is probably similar between cities. Nevertheless, when explored separately, both factors (cities and habitats) had significant effects on the community structure (Table 1a); this is supported by a clear separation between the habitats in the ordination analysis where the exotic species *Passer domesticus* (House Sparrow) and *Columba livia* (Rock Pigeon) appear as common inhabitants of urban habitats (Fig. 3).

**Figure 4:**
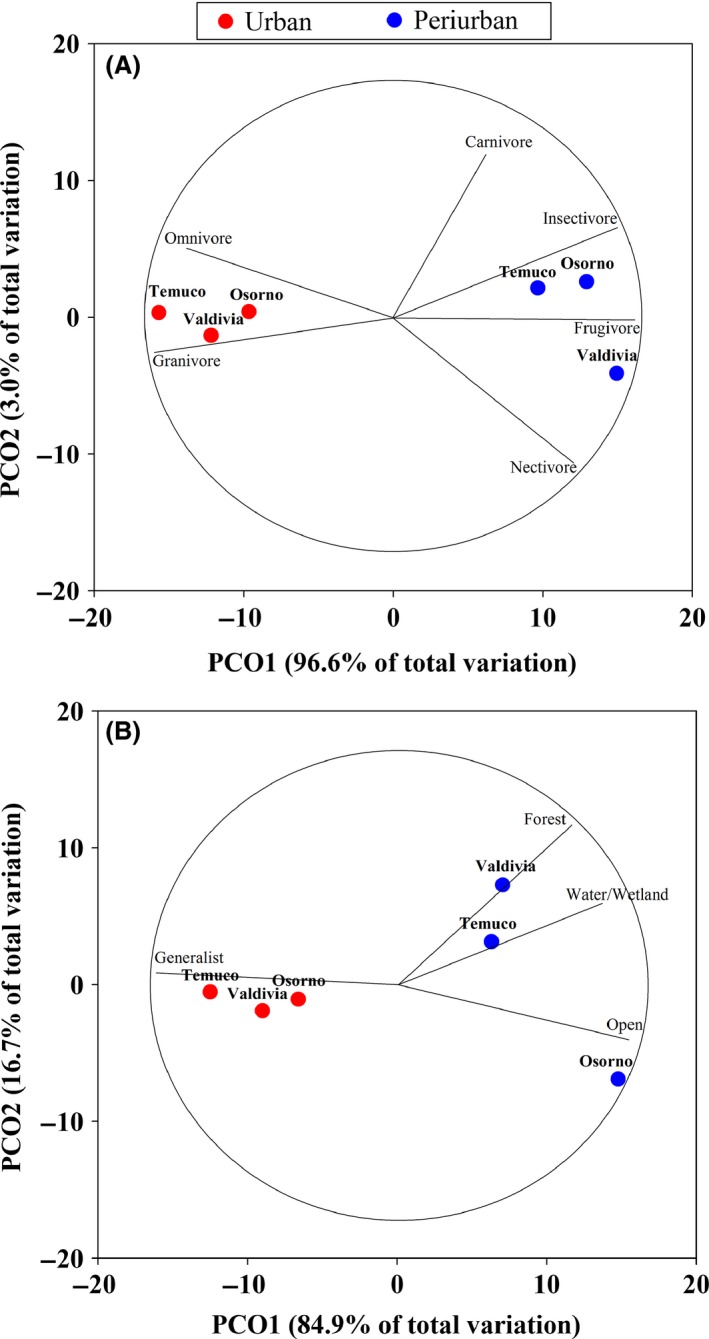
Principal coordinate (PCoA, Bray–Curtis dissimilarity matrices) ordination plot of the bird guild community structure between urban and periurban habitats for Temuco, Valdivia, and Osorno. (A) Diet guilds and (B) preferred habitat guilds. Vectors indicate the correlations >0.75 for each guild.

The statistical analyses revealed that the interaction between city and habitat was significant for preferred habitat guilds (_pseudo_ *F*_(2,487)_ = 5.71; *P*_perm_ = 0.004), but not for diet guilds (_pseudo_ *F*_(2,487)_ = 0.79; *P*_perm_ = 0.518). Despite this, city and habitat, separately, had a significant effect on diet guild structure (Table 1b). These differences were supported by ordination analyses, which showed an evident separation between urban and periurban habitat for both diet (Fig. 4A) and preferred habitat (Fig. 4B) guilds.

The differences in the community structure of urban and periurban habitat for the three cities were mainly due to the changes in the relative contribution of the House Sparrow, Chilean Swallow, and Southern Lapwing according to the SIMPER analyses. House Sparrow abundance explained up to 16% of the total dissimilarities between habitat types for the three cities; the House Sparrow was the most abundant species in the urban habitat, while the Southern Lapwing was the most abundant species in the periurban habitat (Table 2). The average abundance of the Chilean Swallow was highest in the urban habitat of Temuco, while in Valdivia and Osorno the average abundance was similar in both habitat types (Table 2).

**Table 2:** Similarity percentage (SIMPER) routines (cutoff of contributions: 90%) for species that contributed to the dissimilarity between urban (U) and periurban (P) habitats for Temuco (T), Valdivia (V), and Osorno (O) cities, indicating the average abundance per species and the average dissimilarity between habitats

Species	Dissimilarity (% contribution)			Average Abundance					
	TU‐TP	VU‐VP	OU‐OP	TU	TP	VU	VP	OU	OP
House Sparrow^a^	16.06	16.40	16.02	1.34	0.17	1.18	0.23	1.16	0.15
Chilean Swallow	9.43	7.21	8.96	0.54	0.75	0.38	0.34	0.50	0.49
Southern Lapwing	9.30	8.00	10.35	0.28	0.76	0.32	0.44	0.36	0.77
House Wren	6.22	7.05	7.40	0.26	0.44	0.32	0.40	0.35	0.46
White‐crested Elaenia	5.79	7.50	5.66	0.03	0.44	0.11	0.48	0.13	0.37
Austral Thrush	5.40	3.98	3.55	0.12	0.42	0.06	0.27	0.12	0.17
Grassland Yellow‐finch	5.38	3.50	6.44	0.04	0.43	0.12	0.18	0.23	0.38
Black‐faced Ibis	5.00	5.38	7.37	0.15	0.35	0.23	0.25	0.17	0.50
Austral Blackbird	4.28	2.89	2.99	0.05	0.32	0.04	0.24	0.08	0.19
Rock Pigeon^a^	4.00	3.45	5.27	0.32	–	0.24	0.03	0.29	0.11
Black‐chinned Siskin	3.69	3.10	3.38	0.04	0.32	0.09	0.19	0.02	0.23
Rufous‐collared Sparrow	2.68	2.88	1.67	0.02	0.21	0.06	0.19	0.05	0.10
Tufted Tit‐tyrant	2.59	2.60	1.73	0.02	0.22	0.03	0.18	0.04	0.10
Chilean Pigeon	2.34	3.00	1.73	0.01	0.19	0.03	0.20	0.04	0.10
Chilean Mockingbird	1.96	1.47	3.55	0.05	0.14	0.02	0.13	0.07	0.21
Chimango Caracara	1.66	3.13	4.90	0.11	0.04	0.22	0.07	0.23	0.21
Sedge Wren	1.41	–	–	–	0.12	–	–	–	–
Ochre‐flanked Tapaculo	1.37	1.43	–	–	0.12	0.00	0.11	–	–
Green‐backed Firecrown	1.26	3.67	–	–	0.10	0.03	0.25	–	–
Rufous‐tailed Plantcutter	1.23	–	–	–	0.12	–	–	–	–
Des Mur's Wiretail	–	1.84	–	–	–	0.00	0.14	–	–
Long‐tailed Meadowlark	–	1.66	–	–	–	0.02	0.13	–	–
Total	91.05	90.14	90.97						

a Exotic species.

Four of the seven guilds for the diet categories explained 90% of the dissimilarities between urban and periurban habitats. Omnivore, granivore, and insectivore species contributed the most to the dissimilarity between habitats (Table 3). Granivores and omnivores, 12 and five species, respectively, were more abundant in the urban habitat (average abundance = 1.35 and 0.47), while 27 insectivorous species were more abundant in the periurban habitats (average abundance = 1.51, Table 3). When considering preferred habitat guilds, more than 70% of the dissimilarities between urban and periurban areas were explained by the abundance of open and generalist species; generalists were more abundant in the urban habitats, while open species were more abundant in the periurban habitat (average abundance = 1.45 and 1.37, respectively, see Table 3). Despite these general trends, the composition of the guild structures among cities' periurban areas differed (Fig. 4A,B).

**Table 3:** Similarity percentage (SIMPER) routines (cutoff of contributions: 90%) that contributed to the dissimilarity between urban (U) and periurban (P) habitats, indicating the average abundance per guild and the average dissimilarity between habitats

	Dissimilarity (%)	Average abundance	
Guilds	U‐P	U	P
Diet			
Insectivore	29.39	0.95	1.51
Granivore	28.80	1.35	0.97
Omnivore	23.05	0.50	0.47
Frugivore	10.54	0.07	0.31
Habitat			
Open	40.94	0.98	1.37
Generalist	30.61	1.45	1.15
Forest	19.27	0.06	0.36

Fifty‐one native species and three introduced bird species were recorded during this study (Appendix S2). Only 16 species were present in all sample sites; three were present exclusively in urban areas, and 17 were found in periurban areas. Examples of species found in periurban areas include forest specialists such as Chucao and Magellanic Tapaculo and also species of open habitats such as the Fire‐eyed Diucon and Patagonian Sierra‐finch. The most abundant species in the three urban areas were the House Sparrow and the Chilean Swallow, while the Southern Lapwing was most abundant in all three periurban areas.

## Discussion

Although urban habitats can exert both positive and negative effects on bird communities (Chace and Walsh 2006; Evans et al. 2009; van Rensburg et al. 2009), the influence of urbanization is mostly negative. Urbanization is also associated with the loss of many avian species that were present in a particular area before the development (Evans et al. 2009), favouring only a small group of “urban exploiters” (Blair 1996; McKinney 2006). In this study conducted in three cities in Southern Chile, we found a consistent pattern of difference between the community and guild structure of urban and periurban habitats. Furthermore, this pattern of difference was paired with a nonrandom loss of species in the urban areas in relation to the periurban areas, and non‐native species dominated urban communities. These findings also suggest that local biotic homogenization may be occurring in the three cities studied.

Although no statistically significant differences were found in the number of bird species present in both habitat types, the three periurban areas had more species present. This supports previous studies that show that bird richness is negatively affected by urbanization (Chace and Walsh 2006; Chiari et al. 2010; Cam et al. 2011).

Most of the species that were absent in the urban areas are forest specialists. These species, such as the Thorn‐tailed Rayadito, Ochre‐flanked Tapaculo, and Des Mur's Wiretail, were previously described as highly sensitive to fragmentation, patch size, and vegetation structural complexity (Rozzi et al. 1995; Díaz et al. 2005; Willson et al. 2005). However, other forest species such as the Chilean Pigeon and the Slender‐billed Parakeet (Rozzi et al. 1995) were recorded in some urban areas, suggesting that the presence of these species could be due to ecological or behavioral flexibility (Sol et al. 2002; Sih et al. 2011). Nevertheless, other explanations cannot be excluded given that this is among the first studies to evaluate avian communities in Chilean urban areas (Díaz and Armesto 2003; Silva et al. 2015), and it is the only study assessing community structure across cities in South America. It is also noted that the effect of urbanization may have already influenced the adaptation of some species to urban ecosystems.

In this study, two exotic species, *P. domesticus* and *C. livia*, previously described as urban exploiters (González‐Oreja 2011; Przybylska et al. 2012) moderate the urban avian communities. The presence of these exotic species was paired with a low abundance of native bird species consistent with patterns described for other urban areas (e.g., Clergeau et al. 1998 for Quebec and Rennes; Blair 2001 for California and Ohio; and van Rensburg et al. 2009 for South Africa). These results also support previous studies stressing that some biological traits are favoured by urbanization, and this is reflected in the differences in guilds of urban and periurban habitats. Similar to previous studies, we found that urban habitats generally had more granivore, omnivore, and generalist species (Kark et al. 2007; Croci et al. 2008; Conole and Kirkpatrick 2011). At regional scales, it can be seen that there are consistent patterns in the distribution of bird species and guilds despite the different urban development histories. For example, Temuco was founded in 1881, while Valdivia and Osorno were established in 1552 and 1558, respectively. These results suggest that urbanization acts as a filter favouring a similar group of birds and traits, thus promoting biotic homogenization (McKinney and Lockwood 1999; Olden et al. 2004), as it has been found for other biogeographical regions.

One of the main factors affecting avian bird communities is the relationship between sealed surface and green space availability; this is reflected in the significant influence of vegetation cover on species richness, evenness, community structure, diet, and preferred habitat guilds. This relationship is to be expected because green space and its characteristics, such as size, associated vegetation structure, and connectivity, have been identified as key factors influencing bird communities (Kong et al. 2010; Sushinsky et al. 2013).

The functional homogenization of communities could lead to simpler ecosystems with a diminished resilience to environmental change (Olden et al. 2004) and an increased vulnerability to species invasions (Lyons and Schwartz 2001; Marvier et al. 2004). These negative consequences of diversity loss may be exacerbated due to the nonrandom replacement of species with widespread species promoted by urbanization (Sol et al. 2014). Furthermore, diversity loss will likely be even more pervasive ecosystems of high endemism such as the Chilean Winter Rainfall‐Valdivian Forests. As such, studies of this kind are very important in Latin America especially for cities such as Guayaquil, Ecuador; São Paulo, Brazil; and Oruro, Bolivia, which largely span biodiversity hotspots (Pauchard and Barbosa 2013).

A better understanding of the processes and mechanisms regulating avian community patterns and responses to urbanization is necessary to facilitate the implementation of effective conservation and restoration measures (Aronson et al. 2014). However, there is an important biogeographical gap in urban ecosystems research that prevents us from making robust generalizations about the mechanism that generates the changes in community structure due to urbanization. One of the reasons for this is due to the limited availability and access of high‐resolution data for many cities not only in Latina America but also for other regions such as Africa (McHalle et al. 2013) and Asia.

## Conflict of Interest

None declared.

## Supporting information


**Table S1.** Classification of avian species into guild based on their ecological traits.

**Table S2.** Species recorded in the urban and periurban habitats in the Temuco (T), Valdivia (V) and Osorno (O) cities, indicating feeding and preferred habitat guilds.
