# Supplementary material for: Nonrandom filtering effect on birds: species and guilds response to urbanization
Source: Ecol Evol. 2016 May 3;6(11):3711–20. doi: 10.1002/ece3.2144 (PMC4864331; doi:10.1002/ece3.2144)
Supplement: Supplementary file 1 — Table S1. Classification of avian species into guild based on their ecological traits. [file ECE3-6-3711-s001.doc]

**Table S1**

Classification of avian species into guild based on their ecological traits.

| Ecological traits | Guilds | Description |
| --- | --- | --- |
| Diet | Insectivore | diet includes insectivores and small invertebrates |
|  | Granivore | seed eaters |
|  | Omnivore | species spanning three or more guilds |
|  | Carnivore | carnivore, raptors |
|  | Frugivore | fruit eaters |
|  | Nectarivore | nectar eaters |
|  | Carrion | scavengers |
|  |  |  |
| Habitat | Open | species occurs in open areas such as grasslands, playgrounds |
|  | Generalist | species associated to open areas, forest edges and/or forest interior |
|  | Forest | forest interior dwellers |
|  | Water/wetland | species associated to water or wetlands |
