# Supplementary material for: Nonrandom filtering effect on birds: species and guilds response to urbanization
Source: Ecol Evol. 2016 May 3;6(11):3711–20. doi: 10.1002/ece3.2144 (PMC4864331; doi:10.1002/ece3.2144)
Supplement: Supplementary file 2 — Table S2. Species recorded in the urban and periurban habitats in the Temuco (T), Valdivia (V) and Osorno (O) cities, indicating feeding and preferred habitat guilds. [file ECE3-6-3711-s002.doc]

**Table S2**

Species recorded in the urban and periurban habitats in the Temuco (T), Valdivia (V) and Osorno (O) cities, indicating feeding and preferred habitat guilds. For feeding guilds: i, insectivore; c, carnivore; g, granivore; o, omnivore; r, carrion; f, frugivore; n, nectarivore. For preferred habitat guilds: F, forest; G, generalist; O, open; W, water/wetlands. *Exotic species.

|  |  |  |  | Urban | | | Periurban | | |
| --- | --- | --- | --- | --- | --- | --- | --- | --- | --- |
| Linnaean name | Common name | Feeding | Habitat | T | V | O | T | V | O |
| *Agelasticus thilius* | Yellow-winged Blackbird | i | W |  |  |  |  | X |  |
| *Anairetes parulus* | Tufted Tit-tyrant | i | F | X | X | X | X | X | X |
| *Aphrastura spinicauda* | Thorn-tailed Rayadito | i | F |  |  |  | X | X |  |
| *Asio flammeus* | Short-eared Owl | c | O |  | X |  |  |  |  |
| *Callipepla californica** | California Quail | g | O |  |  |  | X | X |  |
| *Chroicocephalus maculipenis* | Brown-hooded Gull | o | O |  | X |  |  |  |  |
| *Cincloides patagonicus* | Dark-bellied Cincloides | i | W | X | X | X |  |  | X |
| *Cistotorus platensis* | Sedge Wren | i | W |  | X | X | X | X | X |
| *Colaptes pitius* | Chilean Flicker | i | O |  |  |  | X |  |  |
| *Columba livia** | Rock Pigeon | o | O | X | X | X |  | X | X |
| *Coragyps atratus* | Black Vulture | r | O |  |  |  |  |  | X |
| *Curaeus curaeus* | Austral Blackbird | g | G | X | X | X | X | X | X |
| *Diuca diuca* | Common Diuca-finch | g | O | X |  |  | X |  |  |
| *Egretta thula* | Snowy Egret | i | W |  |  |  |  |  |  |
| *Elaenia albiceps* | White-crested Elaenia | i | G | X | X | X | X | X | X |
| *Elanus leucurus* | White-tailed Kite | c | O |  |  |  | X |  | X |
| *Enicognathus ferrugineus* | Austral Parakeet | f | F |  | X |  |  |  | X |
| *Enicognathus leptorhynchus* | Slender-billed Parakeet | g | F |  | X |  |  |  |  |
| *Eugralla paradoxa* | Ochre-flanked Tapaculo | i | F |  |  |  | X | X |  |
| *Falco sparverius* | American Kestrel | c | O |  |  |  |  |  | X |
| *Gallinago paraguaiae* | South American Snipe | i | O |  |  |  |  |  | X |
| *Hymenops perspicillatus* | Spectacled Tyrant | i | W |  | X |  | X | X | X |
| *Leptasthenura aegitaloides* | Plain-mantled Tit-spinetail | i | O | X | X |  |  | X |  |
| *Lessonia rufa* | Patagonian Negrito | i | O |  |  |  | X |  |  |
| *Limosa haemastica* | Hudsonian Godwit | i | W |  |  | X |  |  |  |
| *Milvago chimango* | Chimango Caracara | o | G | X | X | X | X | X | X |
| *Mimus thenca* | Chilean Mockingbird | f | O | X | X | X | X | X | X |
| *Molotrus bonairensis* | Shiny Cowbird | i | O |  | X | X | X |  | X |
| *Notoprocta perdicaria* | Chilean Tinamou | o | O |  |  | X |  | X |  |
| *Pardirallus sanguinolentus* | Plumbus Rail | i | W |  |  |  | X |  |  |
| *Passer domesticus** | House Sparrow | g | G | X | X | X | X | X | X |
| *Patagioenas araucana* | Chilean Pigeon | f | F | X | X | X | X | X | X |
| *Phalacrocorax brasilianus* | Neotropic Cormorant | c | W |  |  | X |  |  |  |
| *Phleocryptes melanops* | Wren-like Rushbird | i | W |  |  |  |  | X |  |
| *Phrygilus patagonicus* | Patagonian Sierra-finch | g | O |  |  |  |  | X |  |
| *Phytotoma rara* | Rufous-tailed Plantcutter | o | O |  | X | X | X | X | X |
| *Pteroptochos tarnii* | Black-throated Huet-huet | i | F |  |  |  | X | X |  |
| *Pygochelidon cyanoleuca* | Blue-and-white Swallow | i | W |  |  | X | X |  | X |
| *Scelorchilus rubecula* | Chucao Tapaculo | i | F |  |  |  |  | X |  |
| *Scytalopus magellanicus* | Magellanic Tapaculo | i | F |  |  |  |  | X |  |
| *Sephanoides sephanoides* | Green-backed Firecrown | n | G |  | X | X | X | X | X |
| *Sicalis luteola* | Grassland Yellow-finch | g | O | X | X | X | X | X | X |
| *Sporagra barbata* | Black-chinned Siskin | g | O | X | X | X | X | X | X |
| *Sturnella loyca* | Long-tailed Meadowlark | g | O | X | X | X | X | X | X |
| *Sylviorthorhynchus desmursii* | Des Mur's Wiretail | i | F |  |  |  | X | X | X |
| *Tachycineta meyeni* | Chilean Swallow | i | G | X | X | X | X | X | X |
| *Theristicus melanopis* | Black-faced Ibis | i | O | X | X | X | X | X | X |
| *Troglodites aedon* | House Wren | i | O | X | X |  | X | X | X |
| *Turdus falcklandi* | Austral Thrush | o | G | X | X | X | X | X | X |
| *Vanellus chilensis* | Southern Lapwing | i | O | X | X | X | X | X | X |
| *Veniliornis lignarus* | Striped Woodpecker | i | O |  |  |  | X |  |  |
| *Xolmis pirope* | Fire-eyed Diucon | i | O |  |  |  | X | X |  |
| *Zenaida auriculata* | Eared Dove | g | G | X | X |  | X |  |  |
| *Zonotrichia capensis* | Rufous-collared Sparrow | g | O | X | X | X | X | X | X |
| Total number of species |  |  |  | 21 | 29 | 25 | 35 | 34 | 30 |
